# High-resolution optical coherence tomography in pathology of the vitreomacular interface

**DOI:** 10.1016/j.ajoc.2025.102432

**Published:** 2025-09-18

**Authors:** Lorenzo Ferro Desideri, Karin Paschon, Ines Schumacher, Nicola Sagurski, Yousif Subhi, Janice Roth, Martin Zinkernagel, Rodrigo Anguita

**Affiliations:** a Department of Ophthalmology, Inselspital, Bern University Hospital, University of Bern, Freiburgstrasse 15, CH-3010, Bern, Switzerland; b Bern Photographic Reading Center, Inselspital, Bern University Hospital, University of Bern, Bern, Switzerland; c Graduate School for Health Sciences, University of Bern, Switzerland; d Department of Ophthalmology, Rigshospitalet, Glostrup, Denmark; e Department of Clinical Medicine, University of Copenhagen, Copenhagen, Denmark; f Department of Clinical Research, University of Southern Denmark, Odense, Denmark; g Moorfields Eye Hospital NHS Foundation Trust, London, UK; h Department for BioMedical Research, University of Bern, Murtenstrasse 24, CH-3008, Bern, Switzerland

**Keywords:** High-resolution OCT, Vitreomacular interface, OCT biomarkers, Macular hole, Epiretinal membrane

## Abstract

**Aim:**

This study investigates the diagnostic capabilities of high-resolution optical coherence tomography (HR-OCT) compared to spectral domain optical coherence tomography (SD-OCT) in detecting detailed microstructural changes in vitreomacular pathology.

**Methods:**

This was a prospective cross-sectional study of eyes with vitreomacular interface disease. We included patients with epiretinal membrane (ERM), macular hole (MH), lamellar hole (LH), and vitreomacular traction (VMT). Each patient underwent a comprehensive ophthalmic exam followed by retinal imaging with both SD-OCT and HR-OCT. Images were analyzed for the presence of key biomarkers and the two OCT modalities were compared.

**Results:**

The study cohort consisted of 18 patients with a mean age of 66 years (SD 8.9) and (61.1 %) had biological male sex. HR-OCT provided a superior subcellular view, including superior identification of rod cell nuclei in the outer nuclear layer (ONL) and ganglion cell layer (GCL) and enhanced visualization of biomarkers such as the “cotton ball sign” coupled with IZ disruption (33.3 % vs 5.6 % for HR-OCT and SD-OCT groups, respectively, p = 0.0042). Hyporeflective dots in the ONL, indicative of rod cell nuclei, were seen in 88.9 % of HR-OCT cases but were completely undetectable with SD-OCT (p < 0.0001). Inter-grader reliability was strong, with Cohen's Kappa values for most biomarkers ranging from 0.78 to 0.89.

**Conclusions:**

HR-OCT significantly improves the detection of subcellular features and biomarkers in vitreomacular interface disorders. This device could enhance early diagnosis and monitoring of vitreomacular diseases, with potential correlations to functional outcomes.

## 1 Introduction

Vitreomacular interface diseases include a range of disorders that can lead to progressive visual impairment and structural alterations of the macula. Among them are epiretinal membrane (ERM), lamellar macular hole (LH), full-thickness macular hole (MH), and vitreomacular traction (VMT).^1^ The diagnostic and clinical management of these syndromes increasingly rely on optical coherence tomography (OCT) imaging, which enables a non-invasive visualization of retinal microstructures and provides insight into the pathophysiology of vitreomacular interface-related changes.^1^ In fact, several OCT-based studies have focused on imaging biomarkers, including the preservation of the outer nuclear layers preservation, the integrity of the ellipsoid zone (EZ)/external limiting membrane (ELM) and the condition of the inner retinal layers, correlating these features with clinical outcomes in these patients^2^, ^3^, ^4^

Recent advancements in OCT technology, particularly the development of high resolution (HR) -OCT, have allowed for increased axial resolution, offering potentially improved *in vivo* detailed visualization of retinal microstructures. In contrast with standard OCT with a resolution of approximately 7 μm, HR-OCT achieves around 3 μm, which may reveal finer details of the vitreoretinal interface.^5^^,^^6^ Additionally, on the HR-OCT, power was augmented from 1.2 mW to 2.2 mW, bringing improvement to the contrast of the images.^7^

While initial studies have described the promising application of HR-OCT in some macular diseases, such as intermediate age-related macular degeneration (AMD), central serous chorioretinopathy), to date no evidence has been provided regarding vitreoretinal diseases specifically.^7^^,^^8^

Our study aims to investigate the possible application of HR-OCT in pathologies from the interface vitreomacular and compare it with the current standard of care. Furthermore, it focuses on the potential advantage of this newer device in describing OCT biomarkers associated in those disorders.

## 2 Methods

### 2.1 Study design and participants

This cross-sectional study included patients clinically diagnosed with ERM, MH, LH, or VMT, conducted at the Inselspital, Bern University Hospital, Switzerland. The study was conducted in compliance with the Swiss Human Research Act, ICH Good Clinical Practice guidelines, and the Declaration of Helsinki, with authorization from the local ethics committee (ID-2023-00768). Written informed consent was obtained from all participants.

Inclusion criteria were a confirmed diagnosis of VMT syndrome as determined by a complete ophthalmic examination, including fundus biomicroscopy and OCT. Exclusion criteria were defined as co-existing retinal and macular pathologies, prior retinal surgery, any condition that could obscure OCT interpretation, such as media opacities, and any possible systemic disease potentially affecting macular status.

During the same day, all the study subjects underwent a complete ophthalmological visit and were imaged with both OCT modalities (Standard spectral domain (SD)-OCT and HR-OCT). OCT scans were acquired during the same part of the day (morning) to rule out possible confounding factors like retinal and choroidal thicknesses variations.^9^

### 2.2 OCT imaging protocol

OCT imaging was conducted using both the Standard Spectralis HRA-OCT ((Heidelberg Engineering, Heidelberg, Germany) and the High-Resolution Spectralis OCT systems, research purpose only and not commercially available at the moment (HR-OCT (Heidelberg, SPECTRALIS® High-Res OCT- DMR001). For each modality, the scan area was centered on the fovea, covering a 20° × 20° field of view. The Standard Spectralis HRA-OCT captured OCT volumes measuring 5.90 mm × 5.75 mm × 1.92 mm, with a scan protocol consisting of 49 B-scans at a resolution of 496 a-scans per B-scan. To improve image stability and minimize variations from patient fixation, each scan utilized 25 automatic real-time (ART) frames, ensuring high-quality, consistent captures.

The HR-OCT protocol mirrored that of the standard modality but offered a refined axial resolution of 3 μm, compared to the 7 μm resolution in the Standard OCT. This was achieved with a high-density scan of 1024 a-scans per B-scan, enhancing the ability to visualize finer retinal microstructures. Both OCT modalities employed identical field of view and ART frame settings to ensure comparability of the images. Scans with suboptimal image quality or significant artifacts were excluded from the analysis to maintain data integrity.

### 2.3 Qualitative and statistical analysis

OCT images from both modalities were evaluated for biomarkers, including disruptions in the ellipsoid zone (EZ), external limiting membrane (ELM), interdigitation zone (IZ), as well as features such as intraretinal cysts, epiretinal glial tissue, hyporeflective dots in the outer nuclear layer (ONL) interpreted as rod cell nuclei, and hyporeflective dots in the ganglion cell layer, interpreted as ganglion cell nuclei, as demonstrated in the previous study published by our group.^6^ Other biomarkers investigated were the ‘cotton ball’ sign with IZ focal impairment, the posterior hyaloid visualization, and the gross disruption of the inner retinal layers.^10^

Three experienced ophthalmologists (LFD, JR and RA) independently graded each image set, and inter-grader agreement was evaluated.

### 2.4 Statistical analysis

Statistical analyses were conducted using the Statistical Package for Social Sciences (SPSS), version 15.0. A Shapiro-Wilk test was applied to assess the normality of all variables. Continuous variables were presented as mean ± standard deviation (SD), while categorical variables were represented as percentages. For associations between categorical variables, Fisher's exact test was employed, given the normal distribution of data. Inter-grader agreement was measured using Cohen's Kappa coefficient, adjusted to account for multiple raters. Kappa values, along with 95 % confidence intervals, were calculated to provide precision for agreement estimates.

A power analysis was performed to determine the adequacy of the sample size. Statistical significance was defined as p < 0.05.

## 3 Results

### 3.1 Demographic and clinical characteristics

Eighteen patients were included in this study, with a mean age of 66.0 years (SD 8.9) and 61.1 % (n = 11) males and 38.9 % (n = 7) females. Study subjects were diagnosed with ERM in 50 % of the cases (n = 9), LH in 27.8 % (n = 5), full MH in 16.7 % (n = 3) and VMT in 5.6 % (n = 1). Most of the cohort was pseudophakic (77.8 %, n = 14), while 22.2 % (n = 4) were phakic. Demographic features are summarized in the table (Table 1).

**Table 1:** Demographic and clinical features of the patients with vitreomacular traction syndromes.

Demographic and clinical feature	
Mean Age (SD)	66.0 years (8.9)
Gender	
Males n, (%)	11
Females n, (%)	7
Laterality	
Right n, (%)	10
Left n, (%)	8
Lens status	
Phakic n, (%)	4
Pseudophakic n, (%)	14
Diagnosis	
ERM n, (%)	9 (50.0)
MH n, (%)	3 (16.7)
LH n, (%)	5 (27.8)
VMT n, (%)	1 (5.6)

ERM = epiretinal membrane; LH= Lamellar hole; MH = Macular hole; VMA= Vitreomacular Adhesion.

### 3.2 High-resolution vs. standard spectral-domain OCT

In qualitative analysis, disruption of the EZ was observed in 27.8 % (n = 5) of cases with HR-OCT, while standard OCT identified this feature in 22.2 % (n = 4) of cases (p = 0.148). Similarly, ELM disruption was found in 38.9 % (n = 7) of cases vs the 33.3 % (n = 6) and IZ disruption in 83.3 %/n = 15) and 66.7 % (n = 12) of the patients in the HR-OCT and standard OCT, respectively (p = 0.1204 and p = 0.248). Intraretinal cysts were equally detected by both OCT modalities in 88.9 % of cases (n = 16), as was the presence of epiretinal glial tissue, observed in 38.9 % (n = 7) of the cases with both High-Resolution and standard OCT (p = 1.0).

**Fig. 1:**
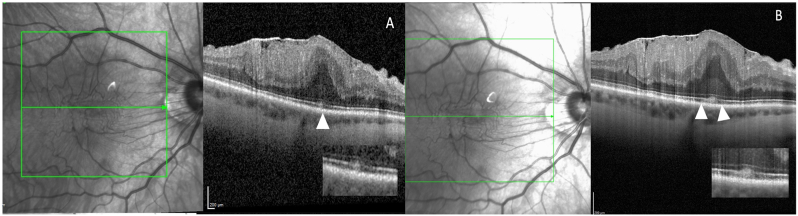
**‘Cotton ball sign’ in a patient with epiretinal membrane.** A standard OCT scan reveals the presence of a small hyperreflective vitelliform material in the foveola between the ellipsoid zone and external limiting membrane without clear impairment of interdigitation zone (IZ) (white arrowhead) B) In HR-OCT beside the hyperreflective material it is possible to identify focal disruption of the IZ in the area around the lesion, probably due to mechanical forces compressing and impairing this thin retinal structure (white arrow heads). Moreover, in the outer nuclear layers an hyperreflective halo around the cotton ball is more clearly distinguishable in comparison with the standard acquisition.

**Fig. 2:**
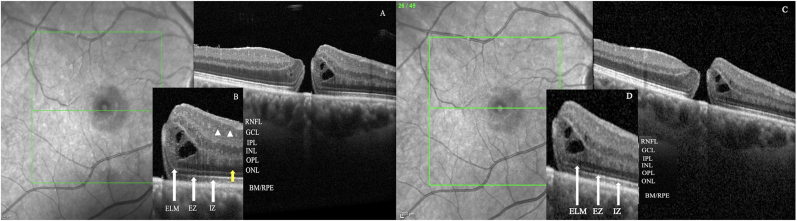
**HR-OCT in a patient with full-stage macular hole** A) The full-retina defect can be appreciated in the OCT scan with intraretinal cysts and no visible disruption in the outer retinal layer on the edge of the hole. B) In the magnified image all the retinal layers can be identified and the ganglion cell nuclei in the GCL layer s roundish hypo reflective spots (white arrow heads) and similarly subcellular detail is provided in the outer nuclear layer with the possible identification of the rod cell nuclei (yellow arrow). In the standard OCT scan subcellular details cannot be distinguished even in the magnified image (C, D).

**Fig. 3:**
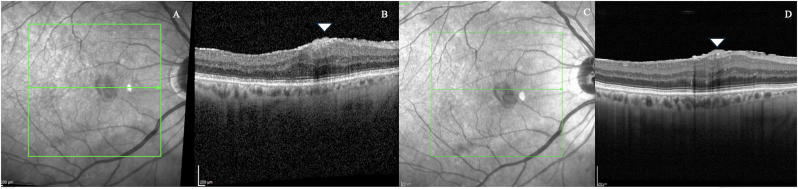
**High-resolution OCT in epiretinal membrane characterization**. Standard infrared reflectance (A) and B-scan OCT (B) images show the presence of an epiretinal membrane (white arrowhead) with limited delineation of its structural components. The corresponding HR-OCT infrared (C) and OCT scan (D) more clearly define the epiretinal tissue, with enhanced contrast and layering. In particular, the HR-OCT highlights differences in hyperreflectivity suggestive of glial proliferation and potentially macrophagic cellular components (white arrowhead), which are less distinguishable in the standard acquisition.

Interestingly, the ‘cotton ball sign’ with focal IZ interruption were more frequently detected by HR-OCT, occurring in 33.3 % (n = 6) of the patients with VMT, compared to 5.6 % (n = 1) with standard OCT (p = 0.042) (Fig. 1). The novel OCT device identified also hyporeflective dots within the ONL in 88.9 % (n = 16) of cases, whereas standard OCT did not detect this feature in any patient (p < 0.0001). Furthermore, hyporeflective dots in the ganglion cell nuclei were observed in 94.4 % of cases on HR-OCT, contrasting with a detection rate of only 16.7 % (n = 3) on standard OCT (p < 0.001) (Fig. 2). (see Fig. 3)

Inter-grader reliability across the three independent graders was robust, with Cohen's Kappa values for most biomarkers ranging between 0.78 (95 % CI: [0.72, 0.84]) and 0.89 (95 % CI: [0.83, 0.94]), indicating substantial agreement (Table 2).

**Table 2:** Comparison in the qualitative imaging biomarkers analysis and inter-grader reliability between the High-Resolution and standard OCT device.

OCT Biomarker	High-Res OCT n (%)	Standard OCT n (%)	P-Value	Cohen's Kappa (95 % CI)
Disrupted EZ	5 (27.8)	4 (22.2)	0.148	0.78 [0.72, 0.84]
Disrupted ELM	7 (38.9)	6 (33.3)	0.1204	0.82 [0.76, 0.88]
Disrupted IZ	15 (83.3)	12 (66.7)	0.248	0.85 [0.79, 0.90]
Intraretinal cysts	16 (88.9)	16 (88.9)	1.0	0.89 [0.83, 0.94]
Epiretinal Glial Tissue	7 (38.9)	7 (38.9)	1.0	0.86 [0.80, 0.91]
Hyporeflective dots in ONL	16 (88.9)	0 (0)	<0.0001	0.87 [0.81, 0.92]
Hyporeflective areas ganglion cell nuclei	17 (94.4)	3 (16.7)	<0.001	0.83 [0.77, 0.89]
‘Cotton ball sign’ with focal IZ interruption	6 (33.3)	1 (5.6)	0.042	0.84 [0.78, 0.89]
ELM continuity edge of MH	1 (5.6)	3 (16.7)	0.6026	0.80 [0.74, 0.86]
Posterior hyaloid visualization	6 (33.3)	4 (22.2)	0.4567	0.81 [0.75, 0.87]
Gross disruption of inner retinal layers	3 (16.7)	2 (11.1)	0.2323	0.78 [0.72, 0.84]

CI= Confidence Interval; ELM = External Limiting Membrane; EZ = Ellipsoid zone; IZ= Interdigitation zone; MH = Macular Hole; ONL= Outer nuclear layer.

## 4 Discussion

This study shows the potential diagnostic value of HR-OCT in capturing microstructural changes in vitreomacular interface disorders compared to standard OCT. HR-OCT provided a more detailed visualization of specific biomarkers such as the ‘cotton ball’ sign with locally disrupted IZ and a better description of retinal structures at cellular and subcellular level, namely the cell nuclei at the ganglion cell layer and the ONL.

Nowadays, a crucial key point in the diagnostic framework of patients with vitreomacular diseases is represented by the early identification of OCT biomarkers associated with the clinical course and functional outcomes of these retinal diseases^11^; although several studies reported the presence of certain OCT biomarkers especially in the outer nuclear layers correlating with disease stage and visual acuity,^12^ the novel HR-OCT device has been reported in other retinal diseases to provide a deeper insight in the visualization of retinal layers and microanatomical details.^5^^,^^7^^,^^8^ In our study the cotton ball sign, defined as a hyperreflective area between the EZ and IZ, was observed in association with early alterations at IZ level in 33.3 % of the patients with HR-OCT, compared to 5.6 % with standard OCT. Govetto et *al*. characterized the ‘cotton ball sign’ as an early biomarker of tractional stress in idiopathic ERM, characterized histologically by tightly packed cone photoreceptors and Müller cells, which lead to the formation of structural “bouquet” vulnerable to mechanical forces in VMT.^13^ Ultimately, the traction from the ERM induces disruptions between the EZ and IZ layers, producing the characteristic hyperreflective area noted on OCT.^14^ In this regard, histological studies suggest that Müller cells may be highly concentrated within the central bouquet and contribute to maintaining retinal architecture through complex interactions with the photoreceptors.^15^ We propose that the ‘cotton ball’ sign, along with the early impairment at the IZ level observed with HR-OCT, reflects early structural alterations in Müller cells under traction.^16^ HR-OCT's superior ability to detect these early changes at the IZ level may offer a more precise visualization of the disorganization within the cone photoreceptor area compared to standard OCT. This enhanced detection not only highlights early clinical signs of mechanical stress but also aligns more closely with the histological modifications reported, providing a clearer understanding of the cellular alterations affecting cone bouquet integrity.

These findings, along with the higher (though not statistically significant) rate of IZ disruptions, align with our previous CSC study, where HR-OCT allowed us to detect subtle alterations at the IZ level in chronic cases more effectively than standard OCT.^8^ This reinforces the potential for HR-OCT to play a broader role in retinal imaging, particularly in detecting microalterations in the outer nuclear layer and other retinal structures. Further large-scale studies could validate these insights and clarify their prognostic value for functional outcomes, ultimately enhancing our ability to monitor and predict early changes in patients with VMT.

Furthermore, our findings support previous research demonstrating the advantages of HR-OCT in detecting subtle outer retinal changes at subcellular level. Trough the adoption of this new OCT device, Reche et *al.* revealed in healthy subjects the identification of finer details of the EZ and IZ that are often missed by standard OCT.^6^ Also, in our study we found that HR-OCT can closely mimic histological sections, enhancing visualization of hyporeflective zones in the ONL corresponding to cell nuclei. As already reported in porcine models, these hyporeflective areas correlate with cone photoreceptor nuclei positioned on the apical ONL side, visible due to a lower refractive index than surrounding structures.^17^ Similarly, we demonstrated also in patients with VMT the higher HR-OCT of detecting ganglion cell nuclei in comparison with the standard device, appearing as a roundish hypo reflective are in the GCL layer as already reported.^6^ This increased level of detail allows for a more precise *in vivo* assessment of retinal microstructures and potential cellular pathology markers. Given its enhanced capability, HR-OCT could prove even more valuable in larger cohorts of patients with advanced-stage diseases of vitreomacular disorders, where retinal architecture is more noticeably distorted. Expanding its application in such cases could establish it as a useful biomarker for disease progression aid in predicting functional outcomes in these patients. Furthermore, the possibility to measure thicknesses between the 2 devices, as done in previous studies for other retinal diseases, might further demonstrate superiority of the HR-OCT device versus the standard one.^7^

Our study has some limitations, including the small sample size, the absence of a long follow-up period, and the heterogenous group of included diseases, including LH, ERM and MH. However, the presence of significant differences between the devices in the detection of microstructural differences in such a smaller group represents a promising basis for future research involving larger samples.

In conclusion, HR-OCT provides detailed visualization of microstructural changes in the pathology of the vitreomacula interface, offering the opportunity to identify novel biomarkers and explain sub-cellular level alterations associated with visual outcomes in the patients. Its broader application in clinical practice may refine not only the diagnostic precision, but also the prognostic assessment of morphological and functional outcomes in the spectrum of vitreomacular interface diseases.

## CRediT authorship contribution statement

**Lorenzo Ferro Desideri:** Writing – original draft, Methodology, Project administration, Conceptualization, Visualization, Investigation, Resources, Formal analysis. **Karin Paschon:** Investigation, Data curation, Methodology, Formal analysis. **Ines Schumacher:** Resources, Funding acquisition, Conceptualization, Methodology, Data curation. **Nicola Sagurski:** Formal analysis, Project administration, Data curation. **Yousif Subhi:** Supervision, Conceptualization, Software. **Janice Roth:** Supervision, Methodology, Conceptualization, Software, Formal analysis. **Martin Zinkernagel:** Writing – review & editing, Supervision, Conceptualization, Validation, Data curation. **Rodrigo Anguita:** Methodology, Funding acquisition, Writing – review & editing, Validation, Project administration, Visualization, Supervision, Investigation, Conceptualization.

## Patient consent

A written consent to publish case details was obtained for all the patients enrolled in the case series.

## Authorship

All authors attest that they meet the current ICMJE criteria for Authorship.

## Funding

No funding or grant support.

## Declaration of competing interest

The authors declare that they have no known competing financial interests or personal relationships that could have appeared to influence the work reported in this paper.
